# Proceedings from the Consortium for Technology & Innovation in Pediatrics (CTIP) 2025 annual pediatric device innovation symposium

**DOI:** 10.1186/s12919-026-00402-3

**Published:** 2026-10-12

**Authors:** Juan Espinoza, Grzegorz Zapotoczny, Bianca Riello, Nadine Afari, Madison Christmas, Jordan Cashwell, Melissa Bent, Yaniv Bar-Cohen

**Affiliations:** 1 Stanley Manne Children’s Research Institute, Ann & Robert H. Lurie Children’s Hospital of Chicago, BOX 205, 225 E. Chicago Avenue, Chicago, IL 6061 USA; 2 Children’s Hospital Los Angeles, Los Angeles, CA USA

**Keywords:** CTIP — Consortium for Technology & Innovation in Pediatrics, FDA — U.S. Food and Drug Administration, MDCTU — Medical Device Clinical Trials Unit, PDC — Pediatric Device Consortia (FDA program)

## Abstract

The 2025 CTIP Pediatric Device Innovation Symposium in Chicago, IL, convened a multi‑stakeholder community of clinicians, entrepreneurs, regulators, investors, and families to examine how pediatric medical devices traverse the “last mile” from regulatory clearance to real‑world adoption. The program featured a keynote fireside chat with Kolaleh Eskandanian, PhD, followed by four focused panels: (1) procurement, reimbursement, and clinical adoption barriers within children’s hospitals; (2) pathways for licensing, M&A, and corporate accelerator partnerships; (3) lived regulatory experiences of CTIP portfolio companies across FDA programs; and (4) investing in pediatrics beyond traditional venture capital. A portfolio showcase highlighted five CTIP companies (WearableDose, Luminoah, Makani Science, Madison Scientific, Renata Medical), with an Audience Choice Award recognizing standout innovation. Cross‑cutting themes included the need for earlier engagement with supply‑chain and finance stakeholders at hospital systems; clear value propositions that link device benefits to total cost of care; creative coding and payer‑evidence strategies; right‑sized quality systems and evidence generation for small pediatric markets; and diversified, mission‑aligned capital stacks (family offices, foundations, strategic partners, and non‑dilutive funding). This manuscript synthesizes session discussions, draws actionable lessons for innovators and health‑system decision‑makers, and outlines near‑term priorities for accelerating safe, effective pediatric device availability and access.

## Introduction

Children remain underserved by a medical device ecosystem built for adults. Most technologies are designed, tested, and labeled for adult populations, often leaving pediatrics to rely on off-label use or legacy equipment. Small and heterogeneous populations, developmental change, and pediatric risk–benefit profiles complicate evidence generation and, in turn, regulatory and reimbursement decisions. Even after FDA clearance, adoption can stall in the “last mile” where misaligned incentives and low volumes magnify friction: value-analysis committees, supply-chain and Group Purchase Organization (GPO) processes, training, and payer coverage and coding.

To address this gap, the US Food and Drug Administration (FDA) has developed a number of programmatic and regulatory initiatives, including creating the Pediatric Device Consortia (PDC) Program. Legislation passed by Congress in 2007 established funding for the PDCs to help stimulate projects to promote the development and availability of pediatric medical devices. This legislation was re-authorized as part of the FDA Safety and Innovation Act of 2012, in the FDA Reauthorization Act of 2017, and the Food and Drug Omnibus Reform Act of 2022. The FDA’s Pediatric Device Consortia (PDC) program funds hubs that provide end-to-end support: need finding, human factors, prototyping, regulatory strategy, clinical studies, reimbursement, business development, and partnerships [1].

## About CTIP

CTIP is one of the five currently FDA-funded PDCs dedicated to the translation and commercialization of pediatric medical devices (PMDs). Now in its third cycle of FDA funding, CTIP has expanded from a Southern California hub (2013–2018) to the Midwest (2018–2023), and today serves as one of the nation’s largest dedicated networks for pediatric medical device innovation. With hubs in Chicago and Los Angeles, CTIP now unites 25 partner organizations across eight states. To date, CTIP has supported more than 220 pediatric device projects, contributed to the successful market entry of 33 devices, and helped portfolio companies raise over $500 million in combined capital, including $40 million in federal non-dilutive grants.

CTIP hosts an annual Pediatric Device Innovation Symposium as an opportunity to bring together the MedTech community and focus on critical issues in pediatric medical device innovation. The 2025 symposium agenda as seen in Table 1 covered critical execution priorities across four strategic domains: procurement and reimbursement infrastructure, strategic partnerships (licensing, M&A, and accelerators), lived regulatory experiences from CTIP portfolio companies, and diversified capital strategies beyond traditional venture funding. The agenda also featured a portfolio showcase highlighting near term commercial innovations. Strategic networking sessions enabled CTIP’s leadership team to facilitate targeted introductions between founders, investors, and partnership prospects. These proceedings distill actionable lessons for innovators and health-system decision-makers and outline next priorities to accelerate equitable, routine use of safe, effective pediatric devices.

**Table 1:** CTIP symposium agenda for Friday August 14, 2025

11:00 am	*Open Networking and Exhibitor Showcase*
1:00 pm	*Welcome (Bianca Riello, MEng; and Pat Seed, MD, PhD)*
1:05 pm	*Opening Remarks (Juan Espinoza, MD)*
1:10 pm	*Keynote: Uncommon Routes: Shaping Purpose‑Driven Innovation in Complex Environments (Kolaleh Eskandanian, PhD)*
1:30 pm	*Panel 1: The Last Mile Problem: Getting Pediatric Devices into Hospitals, Clinics, and Patient Hands*
2:00 pm	*Panel 2: Industry Partnerships: Licensing, M&A, and Corporate Accelerators*
2:30 pm	*Panel 3: Lived Regulatory Experience of CTIP Portfolio Companies*
3:00 pm	*Networking Break*
3:30 pm	*Panel 4: Investing in Pediatrics: Beyond Venture Capital*
4:00 pm	*CTIP Portfolio Showcase*
5:00 pm	*Closing Remarks; Networking & Happy Hour*

The Symposium was structured as a one-day, hybrid event designed to facilitate multi-stakeholder dialogue. The format included a plenary keynote session, followed by four distinct panel discussions, and concluded with a portfolio showcase. The proceedings were compiled using a comprehensive methodology, drawing upon verbatim transcripts, preparatory documentation from each session, and supplementary post-event materials. Attendance comprised a multi-stakeholder community, including clinicians, founders, investors, regulators, payers, and supply chain representatives.

## Opening remarks

Juan Espinoza, Executive Director and Principal Investigator of CTIP and Chief Research Informatics Officer at Ann & Robert H. Lurie Children’s Hospital of Chicago, opened the symposium by thanking Pat Seed and Bianca Riello for launching the event’s program and recognizing the CTIP team whose planning and logistics enabled the event. He framed the day around a persistent reality in U.S. (and global) MedTech: most devices are conceived, engineered, and labeled for adults. As a result, pediatric technologies often trail adult solutions by a decade or more, and children are frequently treated with devices used off‑label, with limited pediatric safety and effectiveness data—an “unfortunate off‑label use” pattern that produces inconsistent benefit–risk profiles and inequitable access. Espinoza noted that the innovation gap is most acute for the youngest (infants and neonates) and the sickest children.

He emphasized that this gap is fixable when devices are designed, evaluated, and labeled for children from the outset. The primary obstacles, he argued, are well understood: (1) scientific and engineering—children’s physiology and neurodevelopment change over time, demanding specialized materials, miniaturization, and longitudinal human‑factors considerations; (2) ethical—children are a protected class, requiring careful attention to consent/assent and the parent–child dyad in research; (3) regulatory—unlike drugs, there are no pediatric‑specific FDA device testing requirements or pathways; and (4) financial—chronic under‑investment in pediatrics and heterogeneous reimbursement create uncertain business cases. Development costs can range from roughly $30 million for many Class I/II devices to $200 million–$300 million for complex Class III devices, typically over 3 to > 10 years, while coverage and payment (Medicaid and commercial) remain variable and unpredictable.

Espinoza situated CTIP’s work within the FDA’s Pediatric Device Consortia (PDC) program, established by Congress in 2007 to create centers of excellence that de‑risk early pediatric device innovation. CTIP began in 2011 as an internal grant between Children’s Hospital Los Angeles and the University of Southern California, received its first FDA grant in 2013, was renewed in 2018 with expansion across the West Coast (eight partner organizations), and in 2023 transitioned to Lurie Children’s with a third FDA grant. Today, CTIP operates a multi‑institution network of 26 organizations across eight states.

He reiterated CTIP’s mission: to advance pediatric medical devices from concept to commercialization. CTIP offers non‑dilutive support (no equity or IP claims) and uses a portfolio‑driven approach to actively source and manage innovations. Support spans direct services (regulatory strategy, hospital integration, business development, partnerships), indirect funding (purchasing specialized services for companies), and direct device funding (non-dilutive grants or awards), complemented by education, events, advocacy, and research via the Medical Device Clinical Trials Unit, anchored in a total product lifecycle perspective.

## Keynote fireside chat | uncommon routes: shaping purpose‑driven innovation in complex environments

- *Speaker: Kolaleh Eskandanian, PhD (Chief Strategy & Innovation Officer; Head of Pediatrics & Special Populations, Compremium AG)*
- *Moderator: Juan Espinoza, MD (Executive Director, CTIP)*

The keynote fireside chat traced Dr. Eskandanian’s “anything‑but‑ordinary” route to pediatric MedTech leadership and distilled lessons for building resilient innovation ecosystems in complex, multi‑stakeholder environments. The discussion emphasized how purpose‑driven leadership can align diverse incentives across regulators, funders, clinicians, and industry to accelerate safe, effective pediatric device availability.

Origins and professional path. Trained in physics and mechanical engineering, Dr. Eskandanian began her career in telecommunications and strategy consulting before being pulled into healthcare through work on adverse‑event reporting and research oversight. This translational pivot—motivated by a desire to solve consequential problems—ultimately led to leadership roles at Children’s National Hospital and, more recently, to her position at Compremium AG. She highlighted how early technical training and operating as one of few women in predominantly male cohorts shaped a leadership style grounded in analytical rigor, voice, and persistence.

Building innovation ecosystems. At Children’s National, Dr. Eskandanian founded Innovation Ventures (the hospital’s commercialization arm) and catalyzed programs that knit together government, academia, and industry. Notable initiatives included the FDA‑funded Pediatric Device Consortium, the BARDA‑supported SPARK accelerator focused on pediatric countermeasures, and JLABS @ Washington, DC—the first life‑sciences incubator on a pediatric hospital campus. A central design principle was to scout the best technologies regardless of institutional origin and to move beyond publication‑centric success metrics toward market impact for children and families.

Aligning diverse stakeholders. Leading initiatives across agencies with distinct cultures—FDA, NIH, BARDA, ARPA‑H, and the European Commission—requires clear articulation of a shared “North Star,” early demonstration of value, and mechanisms that let each party meet its own metrics. The conversation stressed the practical work of coalition‑building: sequencing quick wins, aligning philosophies, and creating collaboration structures where regulators, funders, and developers can all “win” while staying focused on patient benefit.

Looking ahead: purpose and sustainability in pediatrics. From her vantage point in industry, Dr. Eskandanian underscored persistent market failures in pediatrics (e.g., fragile single‑supplier niches such as pediatric mechanical circulatory support) and argued for durable financing models that blend public incentives, mission‑aligned private capital, and philanthropy. She called for earlier, systematic engagement with payers and health systems, greater reliance on pragmatic and real‑world evidence in small populations, and intentional use of partnerships and accelerators (e.g., PDCs) to de‑risk development. While the path remains challenging, she emphasized that devices designed, evaluated, and labeled for children demonstrably improve safety and equity, and that purpose‑driven teams can bend the curve with disciplined execution.

### Take‑home messages:

- Purpose clarifies decisions in complex environments.
- Ecosystem architecture matters as much as invention.
- Alignment is engineered, not assumed.
- Strategic partnerships accelerate market entry and reduce risk.
- Sustainability requires diversified capital stacks and evidence tailored to pediatric needs.
- Execution excellence differentiates successful innovations from promising prototypes.

## Panel 1 | The last mile problem: getting pediatric devices into hospitals, clinics, and patient hands

### Panelists:

- *Katelyn Storton — Senior Director, Supply Chain, Ann & Robert H. Lurie Children’s Hospital of Chicago*
- *Sallie Guezuraga — Director of Business Development, PedsMrkt*
- *Ryan Nolan — Senior Director, Reimbursement/HEOR/Market Access, PRIA Healthcare*
- *Allen Passerallo — VP, Category Management – Orthopedics, Vizient*
- *Moderator: Juan Espinoza, MD*

Session aim and framing. This session interrogated the “last mile” of pediatric device adoption—the complex set of pathways through supply chain, value analysis, contracting, reimbursement, and clinical workflow that ultimately determines whether innovations reach children. Espinoza underscored that many devices falter post‑market not for lack of clinical promise, but because systems are not designed to move pediatric technologies efficiently to the bedside; he estimated the last‑mile work accounts for the majority of the problem.

Reimbursement hurdles and strategies (Nolan). Coverage—more often than coding or payment—was identified as the dominant barrier for pediatric devices. Startups should be able to articulate the current standard of care and position their technology within the full patient‑journey and payment pathway even in pre‑FDA phases. Because FDA evidence thresholds and payer requirements often diverge, companies should prospectively design studies that yield safety, effectiveness, and value data (e.g., utilization, care‑team time, avoidable procedures) within a single program of evidence. Safety and effectiveness data for the FDA is not enough. To achieve market adoption, the objective is to persuade payers and health systems that the device represents a new standard of care with measurable economic impact.

GPO contracting and value (Passerallo). For GPOs, pediatric volume constraints do not preclude contracts when there is credible clinical differentiation. Vizient relies on medical‑evidence directors with deep literature expertise and on input from aggregation groups of children’s hospitals to validate need. Common mistakes include approaching GPOs as mere intermediaries and underestimating how GPOs convene clinicians and operational leaders to interpret data and streamline administrative burden. Successful engagements lead with evidence and a clear operational value case, not price alone.

Market adoption & value propositions (Guezuraga). PedsMrkt provides a platform for collaboration and builds awareness of new solutions across the pediatric ecosystem. It functions as a convenor among innovators and end users. It is able to help bridge silos within the industry and within institutions by helping clinicians, administrators, and technical support to be able to find and evaluate fit and function of new solutions. It addresses the issues of fragmentation of a niche market through a one-stop location for environmental scans and promotes IP and early solutions to investors and other stakeholders to find economies of scale throughout the value stream.

Collaboration across the ecosystem can help provide visibility to how new solutions improve outcomes for pediatric patients through solution validation. This collaboration can also help frame multiple business models, including value based care, to prove sustainability, and broader definitions of value than reimbursement alone.

Hospital supply chain and clinical champions (Storton). Within children’s hospitals, Value Analysis Committees (VACs)—multidisciplinary groups including infection prevention, administrators, and practicing clinicians—are critical decision points. Pediatric devices frequently stall here due to forecasting viability for small firms, confidence in go‑to‑market plans, and the education and protocol work needed for safe adoption. The single most important success factor is a clinical champion who can articulate value to administrators and peers. Storton noted that standalone children’s hospitals are highly collaborative; innovators should leverage peer‑to‑peer networks and dedicated forums (e.g., reverse expos, technology innovation days) to connect with supply‑chain directors and champions.

Policy levers to speed adoption (cross‑panel). Suggestions included requiring formal data submission for devices marketed off‑label for extended periods to facilitate on‑label pediatric use; process improvements in FDA review to reduce time and cost; a standardized common application or standardized intake instrument for VAC and security reviews to reduce redundant effort; and a pediatric analogue to Medicare’s transitional pass‑through payment to bridge early adoption, recognizing that state Medicaid programs lack comparable mechanisms. Panelists also highlighted the role of Pediatric Device Consortia in pooling resources and orchestrating efficient matches among innovators, hospital systems, and grant funders in a coordinated, accelerator‑style structure, amid a challenging investment climate.

### Key takeaways:

- Treat coverage as the rate‑limiting step; design studies that produce safety, effectiveness, and value evidence from the outset.
- Position for GPOs with credible clinical differentiation and an operational value case; engage aggregation groups of children’s hospitals early.
- Build a clinician–administrator coalition at each site; enthusiasm without implementation authority stalls adoption.
- Prepare for VACs: tailored implementation plans, education, infection‑prevention considerations, and demand forecasts for pediatric SKUs.
- Translate benefits into hospital finance metrics (e.g., length of stay, readmissions, staff time, avoidable procedures) rather than clinical endpoints alone.
- Use pilot deployments as evidence‑generating projects with predefined operational and economic endpoints; disseminate results.
- Advocate for policy tools (standardized review “common apps,” pediatric transitional payments) and leverage PDCs to pool resources and reduce transaction costs.
- Collaboration across the ecosystem is required to gain economies of scale and accelerate innovation development and adoption.

## Panel 2 | Industry partnerships: licensing, M&A, and corporate accelerators

### Panelists:

- *Ken Funahashi, MBA, JD — Partner, Corporate Practice, DLA Piper*
- *Derrick L. Maultsby Jr. — Managing Associate, Frost Brown Todd*
- *Paul Magelli, MBA, MPH — CEO, Independent Board Director, Illinois Ventures*
- *Vincent Thomas, MD, MHA — Senior Safety Officer, Cardiovascular Heart Recovery & Circulatory Restoration, Johnson & Johnson MedTech*
- *Moderator: Diana Joseph, PhD (Corporate Accelerator Forum)*

Strategic Objectives and Framework. This executive panel addressed how pediatric medical device innovators optimize partnerships with strategics, legal, investors, and accelerators to achieve market scale. Core discussions focused on licensing versus acquisition strategies, transaction structuring, organizational alignment, and maintaining mission integrity within current capital and regulatory environments.

Strategic Partnerships and Market Dynamics. Executive polling during the panel revealed consensus that large enterprise organizations consistently play critical roles as customers, development partners, or acquirers across successful commercialization pathways. Timing perspectives varied significantly. Growth advocates highlighted pediatric programs (PDCs and CTIP), non dilutive funding opportunities, and expanding global markets. The prevailing view acknowledged improved market receptivity compared to prior decades while recognizing persistent execution challenges from elevated MedTech failure rates and compressed valuations.

Acquisition versus Licensing Models. From corporate perspectives, acquisitions streamline intellectual property control and accelerate product integration, particularly when internal development capabilities are constrained. For founding medtech teams, premature exits present strategic concerns. However, when scale requirements, quality infrastructure, or market access capabilities become limiting factors, acquisition often provides the most efficient path to patient impact. Panelists emphasized rigorous strategic alignment assessment to prevent post transaction product discontinuation, noting that physician entrepreneurs frequently prioritize sustainable institutional platforms over direct operational control.

Partnership Execution Framework. Successful case studies demonstrated strategic positioning approaches: expanding narrow device concepts into comprehensive platforms or service offerings to maximize addressable markets; incorporating corporate partnership roadmaps into investor presentations detailing target partners and adoption acceleration strategies; developing clinical evidence portfolios addressing both pediatric and broader market applications; and strategically recruiting seasoned commercial leadership. Comparative analyses illustrated bootstrapping limitations within complex device ecosystems while demonstrating how mission aligned strategic and philanthropic capital enables superior exits when combined with robust ecosystem positioning and intellectual property strategies.

Risk Management and Ecosystem Leverage. Panelists described the current federal and economic landscape as characterized by policy uncertainty including trade implications and supply chains, funding volatility, and regulatory transitions alongside opportunities for innovative venture and strategic relationships. Recommendations included maximizing consortium resources through CTIP and Pediatric Device Consortia for partnership development, resource access, and risk mitigation, while approaching entrepreneurship as a sustained strategic initiative requiring organizational resilience, phased capital deployment, and unwavering focus on end user value creation for pediatric populations, caregivers and families.

### Key takeaways:

- Assume a strategic will be involved somewhere on the path (customer, partner, or acquirer); design options for each.
- Calibrate timing expectations: support for pediatrics is growing, but capital is selective and execution remains hard.
- Choose the right path to scale: weigh licensing versus M&A based on capabilities, time‑to‑impact, and IP control; avoid acquirers misaligned with the technology’s mission.
- Engineer fit and evidence: articulate a partner strategy in the deck; generate data packages that speak to pediatric and adult value where appropriate.
- Professionalize early: clinician‑founders should consider experienced CEOs/operators to manage partnerships, regulatory, and investor relations.
- Broaden the value story: reframe devices as platforms/services when credible; expand the total addressable market (TAM) without losing pediatric focus.
- Be realistic about capital needs: bootstrapping is rare in MedTech; use non‑dilutive sources for niche problems and consider out‑licensing.
- Leverage ecosystems: engage CTIP/PDCs and accelerators for matchmaking and de‑risking; cultivate non‑traditional relationships.
- Play the long game: resilience and mission discipline matter; expect multi‑year journeys with staged inflection capital.

## Panel 3 | Lived regulatory experience of CTIP portfolio members

### Panelists:

- *Dan Clark — Founder, Linear Health Sciences*
- *Eason Abbott — Co‑Founder, President, Board Member, Renata Medical*
- *Xina Quan — Co‑Founder & CEO, PyrAmes*
- *Moderator: Grzegorz Zapotoczny, PhD (Director, Medical Device Clinical Trials Unit, CTIP)*

Session aim and framing. This session celebrated recent regulatory milestones among the CTIP portfolio members and offered actionable guidance based on the experience of founders who successfully navigated divergent FDA interactions, expedited programs, and pediatric‑specific clinical evidence generation expectations. The discussion highlighted how regulatory pathways are shaped as much by product class and review team culture as by formal guidance, and how proactive strategy can reduce cycle time and uncertainty.

Linear Health Sciences (Clark). Linear’s Orchid Safety Release Valve for IV lines was accepted to FDA’s Safer Technologies Program (SteP) and received 510(k) clearance for adult use in 2022. Clark indicated that although the device was specifically developed to improve outcomes in children, the FDA’s bar for pediatric-specific labeling was extremely high necessitating a 2-step approach. Thanks to his perseverance and compelling arguments, pediatric use was approved through a subsequent 510(k) submission the following year. Clark emphasized the importance of pediatric labeling in medical devices, and the founder’s role in educating regulators, engaging external clinical experts/KOLs, and “bullet‑proofing” the regulatory strategy to withstand FDA’s scrutiny.

PyrAmes (Quan). PyrAmes develops continuous, non‑invasive blood pressure monitoring systems for babies in the NICU, including extremely low‑birth‑weight infants. The Boppli Monitor was designated a Breakthrough Device, which provided an opportunity for an early FDA engagement. Aligning with the FDA on the device validation strategy is critical, and Quan suggested the company should have focused on discussing study protocol and sample‑size expectations, rather than details of software algorithms, especially when still in development. While participation in the Breakthrough Device sprint discussions was time-consuming and did not fully de-risk device development, it conferred credibility with investors and helped the team prepare for the rigor of 510(k) interactions; resulting in securing 510(k) clearance in 2023.

Renata Medical (Abbott). Renata’s Minima Growth Stent is designed for implantation in neonates and infants and features a dilation mechanism to match child’s growth to eliminate the need for recurring stent-replacement surgeries. Spun out in 2019, the team proactively mapped a multi‑year plan to secure FDA approval; including BPD participation. Abbott characterized their device development journey as a story of effective FDA collaboration to break the long‑standing practice of using adult devices in children off-label. Renata obtained Premarket (PMA) approval in late 2024 which is an unprecedented achievement for a high-risk implantable device.

Cross‑cutting insights. Device sponsors approaching the FDA should remain open-minded as the experiences during review differ widely depending on medical specialty regulations, review panel composition, or even communication styles of individual FDA officers. Across cases, three themes recurred: (i) device validation strategy must not only be responsive to regulatory (safety and effectiveness) requirements, but also meet payor expectations, and align with hospital workflows to facilitate adoption); (ii) early, forthright engagement through pre-submission meetings or sprint discussions (BDP/SteP), and, when necessary, issues escalation to FDA leadership – reduces ambiguity; and (iii) pediatric ecosystem engagement (patient/families, advocacy groups, Pediatric Device Consortia, other device accelerators) improves credibility and reduces time to adoption.

### Key takeaways:

- Approach FDA early to offset heterogeneity: alignment takes time, especially with differing interpretations and communication norms across divisions; document decisions and meeting minutes.
- Educate proactively: bring KOLs to help you emphasize the impact of your technology on pediatric outcomes; close knowledge gaps around pediatric risk profiles and human factors needs.
- Design a holistic evidence generation strategy that supports device’s safety/effectiveness for the FDA and value analysis metrics for payors or hospitals (e.g., workflow, utilization, avoidable events).
- Use FDA’s expedited programs judiciously: BDP and SteP-associated discussions can help with access and alignment but are not guarantees of smoother reviews; manage expectations.
- Escalate when needed: use supervisory reviews/appeals pathways if requests become inconsistent with risk or precedent; stay professional and data‑driven.
- Right‑size studies for pediatrics: negotiate sample sizes, endpoints, and statistical plans suited to small, vulnerable populations; consider pragmatic/RWE designs.
- Leverage the ecosystem: engage CTIP/PDCs for business, clinical, regulatory, and reimbursement strategy support; connect with patients and patient advocacy groups to validate pediatric use case
- Align capital with regulatory milestones: BDP/SteP acceptance can help secure angel investors, while securing the Investigational Device Exemption (IDE) to initiate clinical trials will provide credibility for venture funding.
- Think beyond clearance/approval: research what are the success metrics for the payors and hospital value analysis committees and incorporate them into your evidence generation strategy to minimize the time between the regulatory decisions and commercialization.

## Panel 4 | Investing in pediatrics: beyond venture capital

### Panelists:

- *Adam Heller (Oakwood Circle Ventures LLC)*
- *Kirstie Keller, PhD (Additional Ventures)*
- *Branden Rosenhan, MD (MedMountain Ventures)*
- *Tyler Wanke, MD, MBA, MEM (Wanke Family Office)*
- *Moderator: Madison Christmas (Program Associate, Investment & Growth, CTIP)*

Session aim and framing. With HealthTech venture activity tightening post‑pandemic—especially at the earlier stages—this session examined how pediatric innovators can finance progress when traditional Venture Capital (VC) is selective. Panelists discussed the current deal climate, what makes ventures attractive to institutional investors, and practical pathways to engage non‑VC capital (family offices, angels, physician investors, foundations/DAFs, mission-driven funds, incubators/accelerators).

The current venture climate. HealthTech VC deal activity has decreased since 2021, with the majority of dollars increasingly gravitating towards lower-risk and later-stage companies. Panelists attributed this increased market caution to inflated valuations during the pandemic and delayed investment returns. Furthermore, they identified that reimbursement challenges and hospital budget constraints have made investors more hesitant to invest in the MedTech sector, citing that this increased caution is especially strict for pediatric medical device innovators, as early pediatric deals are required to meet higher standards for novelty, market appeal, and pathway to reimbursement and payment.

What attracts venture capital. Given the competitive market today, companies seeking VC funding must present innovative, game-changing solutions that disrupt the current paradigm, rather than just offering incremental improvements. Panelists noted that investors seek clear connections to revenue‑generating service lines and data packages that demonstrate economic impact, such as cost avoidance, staff‑time savings, and fewer complications. Additionally, they emphasized that VC funds aim to achieve portfolio-level return targets, so realistic exit pathways (M&A/IPO), capital needs, and timelines are crucial to the pitch. Finally, they urged the importance of a concise narrative, warm introductions, and visible enthusiasm from KOLs to help build momentum in interest.

Alternatives beyond traditional VC. Family offices were shared as increasingly important sources of capital for early-stage medical technology (MedTech) companies, with many focusing on mission or impact-driven investments. They are often willing to accept less favorable terms if the deal aligns with solving significant problems. The panelists urged that Angel investors and physician investors may also pursue impact-oriented goals, offering flexible funding and strategic connections that can support these objectives. The panelists discussed several non-dilutive private funding options that prioritize mission, such as grants, recoverable grants, and program-related investments from foundations and donor-advised funds (DAFs), which can enhance credibility in the patient community. Other options mentioned included small business loans and structured non-dilutive instruments. The final pathway mentioned was incubators and accelerators, such as mHUB’s MedTech program and the CTIP and Additional Ventures initiative, which offer capital and guidance to support prototype and product development, as well as access to a broad network of investors.

Finding and engaging alternative funders. The panelists emphasized that securing alternative capital is a “momentum game” that requires persistence and a strategic approach. Recommended tactics include targeted conference participation, systematically mapping comparable companies’ cap tables to identify syndicates and risk appetites, and developing a live database of funders and criteria. Founders should tailor their materials to different investor types and select the most suitable instrument (grant, loan, or equity) based on the stage and risk level. Leveraging CTIP and the Pediatric Device Consortia network can help fill matchmaking gaps with hospitals, strategics, and SBIR or other grant programs. Ultimately, fundraising is a momentum game built on repeated, high-quality interactions.

### Key takeaways

- Demonstrate value + impact: pair differentiated clinical value with a clear coding/coverage/payment plan and quantified economic impact.
- Identify right‑fit capital: align investor type to stage and market size (VC for scale; family offices/DAFs/angels for earlier, mission‑driven bets).
- Design for exit: Define realistic M&A and IPO paths, capital requirements, and timing.
- Broaden the pipeline: use accelerators/incubators and physician investors; combine non‑dilutive tools with equity.
- Network deliberately: pursue warm intros; mine peer cap tables; show KOL advocates; maintain a dynamic funder database.
- Tell a concise story: crisp problem–solution–model framing that resonates with both clinicians and capital partners.
- Leverage ecosystems: engage CTIP/PDCs to bridge to hospitals, payers, and strategics and to navigate grants (e.g., SBIR/STTR).
- Persist: treat fundraising as a momentum process based on reach, measured progress, and disciplined follow‑up.

## CTIP portfolio showcase

### Companies featured:

- *Makani Science*
- *Renata Medical*
- *Luminoah*
- *WearableDose*
- *Madison Scientific*
- *Moderator: Bianca Riello, Meng (Managing Director, CTIP)*

Session aim and framing. The symposium concluded with a curated showcase of five CTIP‑supported companies, designed to introduce founders and their technologies to a cross‑sector audience of clinicians, supply‑chain leaders, strategics, and investors. Attendees voted for an Audience Choice Award ($10,000 in CTIP consulting services), underscoring near‑term applicability and momentum.

CTIP portfolio snapshot. CTIP has evaluated > 600 pediatric device opportunities and maintains an active portfolio of > 100 projects across all device classes. In the most recent five‑year grant cycle, 21 portfolio devices reached the market; 6 were licensed or acquired; 7 received Breakthrough Device designation; and 3 participated in FDA’s Safer Technologies Program (SteP). Since joining CTIP, portfolio companies have collectively raised > $500 M in equity and > $90 M in non‑dilutive funding, with prototype as the most common intake stage.

## Makani Science — Continuous respiration monitoring across the lifespan

### Presenter: Greg Buchert, MD (CEO)

Makani Science is developing the world’s first continuous respiration monitor designed for all ages, from premature infants to adults. This game-changing medical device continuously monitors the breathing pattern waveform and records it in the cloud. Their first-generation device has FDA clearance for measuring respiratory rate and also detects volume breathing patterns and body position, notably without the motion artifact seen in other wireless, wearable respiratory monitors. A second-generation device, currently in R&D, will feature a two-week battery life and also detect heart rate and pulse oximetry.

### Renata Medical — Growth‑capable endovascular solutions for congenital heart disease

#### Presenter: Morey Marshall (Director of R&D)

Renata Medical has developed the Minima growth stent, their first product that received PMA approval at the end of 2024, designed for neonates and infants. It can be implanted using a 4F-equivalent catheter in a day-old baby and has the unique ability to be periodically re-expanded throughout the patient’s entire lifetime as they grow. This stent addresses narrowings, often congenital, or resulting from open-heart surgery, offering a less invasive transcatheter alternative to repeated surgeries that carry high mortality and morbidity rates.

The company is also developing the Lumina shunt system, their second product line, targeting single ventricle disease, specifically as a transcatheter alternative to the third, highly invasive Fontan open-heart surgery. This system aims to provide an appropriately sized device that can be re-expanded as the child grows, avoiding the use of oversized conduits in young children that cannot accommodate adult vessel sizes.

### Luminoah — Closed‑loop enteral nutrition for personalized care

#### Presenter: Neal Piper (Founder & CEO)

Luminoah’s creation was deeply personal, inspired by Neal’s son, Noah, who underwent cancer treatment and faced challenges with enteral feeding devices. Noah is now nine years old and cancer-free, motivating the company to create a brighter future for people needing tube feeding. Luminoah has developed the world’s first closed-loop enteral nutrition delivery device for personalized nutrition in chronic disease care. The system includes the world’s smallest enteral pump (smartphone-sized), a smart nutrition pouch, and a comprehensive data ecosystem. The mobile app provides real-time nutritional data (calories, hydration, weight changes) and integrates with smart scales to show trend lines of calorie input and output. The provider portal, “Luminoah Intelligence,” aims to triage patients with a nutrition score (green, yellow, red) to intervene before malnutrition, a problem associated with a 5 × increase in mortality and a major financial loss to the health system.

### WearableDose — Real‑time in‑vivo radiation dosimetry

#### Presenter: John B. Sanwo (CEO)

WearableDose is developing a real-time wearable smart patch for precision radiation therapy, addressing a critical gap in current clinical practice: the lack of continuous in-vivo dose measurement during treatment. The WearableDose patch electronically detects ionizing radiation, including protons, photons, and neutrons, and streams this data into a secure platform that contextualizes delivered dose across primary and secondary indications. The Dynamic Treatment Planning platform integrates five previously untracked parameters—treatment protocol, cumulative dose, dose fractionation, surface geometry, and application modality—providing clinicians with real-time insight into planned versus delivered dose. By combining in-vivo dosimetry with machine-learning–driven analytics, WearableDose aims to enable more precise targeting, reduced exposure to healthy tissue, and continuous treatment optimization—moving radiation therapy from static assumptions to real-time, patient-specific dose intelligence.

### Madison Scientific — Smart shunt for hydrocephalus with active over‑drainage control

#### Presenter: Tyler Wanke, MD (Co‑Founder & CEO)

Madison Scientific (MadSci) is developing an implantable, sensor-enabled shunt system for the treatment and monitoring of hydrocephalus and other neurologic conditions. The technology builds on the standard neurosurgical shunt by integrating intracranial pressure (ICP) sensing with an electromechanical valve and embedded control logic, enabling real-time pressure monitoring and more precise regulation of cerebrospinal fluid drainage. By distinguishing pathologic pressure changes from transient physiologic events, the system is designed to address over-drainage, a leading contributor to shunt failure and patient morbidity. In parallel, the SmartShunt’s remote monitoring platform provides continuous, non-invasive ICP diagnostics, allowing physicians to track shunt function and intracranial dynamics in real time, outside the hospital – allowing for intervention before failure occurs. Taken together, the system integrates treatment and monitoring within a single implantable platform, representing a paradigm shift in hydrocephalus management.

Closing. Luminoah was selected as the Audience Choice Award winner, receiving $10,000 in consulting services from CTIP. Dr. Espinoza concluded by thanking presenters, attendees, sponsors, and host institutions for their role in strengthening the pediatric medtech ecosystem, followed by a hosted networking reception.

## Reach and impact

The 2025 CTIP Symposium brought together 217 total attendees, including 122 in-person and 95 virtual participants, representing 23 U.S. states and three countries (Table 2). With strong representation from healthcare, industry, research, and startup sectors, the event demonstrated CTIP’s continued ability to foster cross-sector collaboration in pediatric medical device innovation (Table 3). Most attendees hailed from Illinois and California, reflecting CTIP’s regional influence and growing national reach, while the international presence from countries such as England and Switzerland highlighted the consortium’s expanding global footprint. The primary source of awareness (Table 4) was word of mouth, followed by CTIP Social Media.

**Table 2:** Registrants and attendees by region

Region	Registrants n (%)	Attendees (%)
Midwest	163 (57.6%)	102 (57.6%)
West	59 (20.8%)	41 (23.2%)
South	22 (7.8%)	14 (7.9%)
Northeast	28 (9.9%)	17 (9.6%)
International	11 (3.9%)	3 (1.7%)

**Table 3:** Registrants and attendees by sector

**Sector**	**Registrants n (%)**	**Attendees n (%)**
Academia	71 (15%)	35 (12.1%)
Accelerators & Incubators	30 (6.38%)	21 (7.3%)
Design and Manufacturing	27 (5.74%)	18 (6.23%)
Entrepreneur or Startup	76 (16.2%)	54 (18.7%)
Government	1 (0.2%)	1 (0.3%)
Healthcare Provider	45 (9.57%)	21 (7.3%)
Industry	44 (9.36%)	30 (10.4%)
Insurance & Payers	6 (1.28%)	4 (1.38%)
Investors	20 (4.26%)	13 (4.5%)
Patient or Patient Advocate	17 (3.62%)	9 (3.11%)
Philanthropy	7 (1.49%)	5 (1.73%)
Regulatory Professional	7 (1.49%)	4 (1.38%)
Research	66 (14%)	42 (14.5%)
Vendor or Consultant	35 (7.45%)	22 (7.61%)
Other	18 (3.83%)	10 (3.46%)

**Table 4:** Source of awareness

Category	Registrants (n = X)	Attendees (n = X)
	Count (%)	Count (%)
A colleague or friend	106 (33.4%)	72 (36.6%)
CTIP Social media	29 (9.15%)	14 (6.97%)
CTIP Website	14 (4.42%)	11 (5.47%)
CTIP email or newsletter	68 (21.5%)	48 (23.9%)
Lurie Children's email or newsletter	37 (11.7%)	20 (9.95%)
One of the Pediatric Device Consortia	25 (7.89%)	15 (7.46%)
Other	36 (11.4%)	19 (9.45%)
Prefer not to share	2 (0.6%)	1 (0.5%)

Evaluation results reflected overwhelmingly positive feedback, with an average overall rating of 4.8 out of 5 and a Net Promoter Score of 9.6. Attendees praised the relevance of panel discussions, the expertise of speakers, and the practical applicability of the sessions. Nearly all respondents indicated the event length was “just right,” and 93% said they would attend again in 2026. Additionally, two-thirds expressed interest in joining CTIP’s newsletter, showing strong engagement beyond the event itself. Several attendees also noted appreciation for networking opportunities and the mix of academic and industry perspectives that made discussions dynamic, and solution focused.

Looking ahead, attendees suggested incorporating more startup case studies, interactive sessions, and extended networking time at future symposiums. With consistently high satisfaction, a growing international presence, and strong interest in continued partnership and sponsorship, the 2025 CTIP Symposium successfully advanced CTIP’s mission to accelerate pediatric innovation through collaboration, knowledge exchange, and community building.

Data is reported for registrants and attendees that have completed the event registration form; virtual attendees without clear Zoom naming may not be captured.

## Conclusion & looking ahead

The 2025 CTIP Pediatric Device Innovation Symposium advanced the field from problem‑framing to solution development. Across the keynote and four panels, stakeholders converged on practical levers to move pediatric devices from clearance to routine use: co‑designing studies that generate safety, effectiveness, and value evidence in tandem; engaging supply‑chain, value‑analysis, and payer partners early with institution‑specific implementation plans; engineering partnerships (licensing or M&A) that protect mission and prevent post‑deal shelving; and diversifying capital stacks with family offices, foundations/DAFs, angels, and accelerators alongside traditional venture capital. Case studies from CTIP portfolio companies underscored heterogeneity in FDA interactions and the importance of disciplined pre‑subs, judicious use of regulatory programs, and, when needed, escalation pathways anchored in data and precedent. Policy opportunities—standardized procurement processes for hospital review, pediatric transitional payment mechanisms, and stronger pathways for on‑label pediatric use—were highlighted as force multipliers.

CTIP and its partners in the FDA Pediatric Device Consortia program will continue to translate these lessons into tools, matchmaking, and clinical‑trial/RWE infrastructure that de‑risk adoption for children’s hospitals and payers. Our shared aim remains unchanged: through partnership, evidence, and thoughtful commercialization, to shorten the distance between innovation and the children who need it most.

## Data Availability

Data and materials supporting the findings presented in this proceedings article are available from the corresponding author upon reasonable request, subject to applicable institutional and confidentiality requirements.

## Abbreviations

ARPA-H: Advanced Research Projects Agency for Health
BARDA: Biomedical Advanced Research and Development Authority
BDP: Breakthrough Devices Program (FDA)
CRIO: Chief Research Informatics Officer
CTIP: Consortium for Technology & Innovation in Pediatrics
DAF: Donor-Advised Fund
FDA: U.S. Food and Drug Administration
GPO: Group Purchasing Organization
HEOR: Health Economics and Outcomes Research
IDE: Investigational Device Exemption
IPO: Initial Public Offering
JLABS: Johnson & Johnson Innovation – JLABS (incubator)
KOL: Key Opinion Leader
M&A: Mergers and Acquisitions
MDCTU: Medical Device Clinical Trials Unit
NICU: Neonatal Intensive Care Unit
NIH: National Institutes of Health
PDC: Pediatric Device Consortia (FDA program)
PMA: Premarket Approval
R&D: Research & Development
RWE: Real-World Evidence
SBIR: Small Business Innovation Research
SteP: Safer Technologies Program (FDA)
TAM: Total Addressable Market
VAC: Value Analysis Committee
VC: Venture Capital
510(k): Premarket Notification (Section 510(k), FDA)
